# Effect of Vitamin D Supplementation on Intracytoplasmic Sperm Injection Outcomes: A Randomized Double-Blind Placebo-Controlled Trial 

**DOI:** 10.22074/ijfs.2019.5470

**Published:** 2019-01-06

**Authors:** Sara Abedi, Mahboubeh Taebi, Mohammad Hosein Nasr Esfahani

**Affiliations:** 1 Student Research Committee, School of Nursing and Midwifery, Isfahan University of Medical Sciences, Isfahan, Iran; 2 Department of Midwifery and Reproductive Health, School of Nursing and Midwifery, Isfahan University of Medical Sciences, Isfahan, Iran; 3 Department of Reproductive Biotechnology, Reproductive Biomedicine Research Center, Royan Institute for Biotech- nology, ACECR, Isfahan, Iran

**Keywords:** Assisted Reproductive Techniques, Infertility, Pregnancy, Vitamin D

## Abstract

**Background:**

Despite numerous studies indicating an imperative role for reproduction, however, the role of Vitamin D supplementation on outcomes of assisted reproductive techniques remains controversial. This clinical trial was per- formed to evaluate the effect of Vitamin D supplementation 6 weeks prior to intracytoplasmic sperm injection (ICSI) on fertility indices.

**Materials and Methods:**

The present study was a double-blind clinical trial conducted on infertile women was ran- domly allocated into two groups: Vitamin D supplementation (42 participants) and placebo (43 participants). Serum Vitamin D was measured before and six to eight weeks after treatment, on the day of ovum pick up. Results were analyzed using SPSS16 and fertility indices were compared between the two groups.

**Results:**

No significant difference was observed between the intervention and control groups regarding the mean number of oocytes retrieved, percentage mature oocyte, fertilization rate and the rate of good quality embryos (all P>0.05). But, percentages of the individual with suitable endometrium (7-14 mm thickness) were significantly higher in the Vitamin D compared to control group (P=0.011). The rate of chemical (47.6 vs. 25.5%, P=0.013) and clinical pregnancy rate (38.1 vs. 20.9%, P=0.019) were also significantly higher in the Vitamin D compared to control group.

**Conclusion:**

The present study reveals that consuming Vitamin D for 6 weeks prior to ICSI improves quality of endo- metrium, rate of chemical and clinical pregnancy (Registration Number: IRCT2015111124999N1).

## Introduction

Infertility is multifactorial in its origin and is affected by different factors including lifestyle, eating habits or nutrition. Numerous studies have shown that reduce exposure to sunlight and poor eating habits have led to Vitamin D insufficiency and/or Vitamin D deficiency, even in sunny countries among men and women of reproductive age (1) and this phenomenon is considered as one etiology for infertility (2-7).

Vitamin D is a fat-soluble vitamin and is considered as an essential nutrient required for our health. One of the main functions of Vitamin D is to help with the absorbance of calcium and phosphate, and helps building bones and keeps them strong and healthy. It also blocks the release of the parathyroid hormone involved in reabsorption of bone tissue, which makes bones thin and brittle. Considering these functions of Vitamin D, it plays a central role in calcium and phosphate hemostasis and in turn is-needed for the normal mineralization of bone, muscle contraction, nerve conduction, and general cellular function in all cells of the body including cell growth.

Vitamin D receptor (VDR) is a member of nuclear receptor family of transcription factors. It forms a heterodimer with a retinoid-X receptor and binds to hormone response elements on DNA to regulate expression of specific gene products. At post transcriptional level Vitamin D regulates gene expression through microRNA-directed mechanisms (7). VDR is present throughout reproductive axis including endometrial epithelial cells, granulosa, fallopian tube epithelial cells and cells of cumulus oophorus in ovaries (8). Therefore, the reproductive axis is considered as one of the target organs for Vitamin D (9). In this regard, some studies have advocated Vitamin D plays role in the biosynthesis of sex hormones (estrogen and progesterone) and also post fertilization in the process such as implantation (10) and production of human chorionic gonadotropin (hCG) (11). Considering roles of Vitamin D in reproductive biology, numerous studies have shown the association between Vitamin D insufficiency and deficiency with fertility or poor pregnancy outcomes (12, 13). In this regard, Somigliana et al. (14) showed the time to pregnancy is longer in women with Vitamin D insufficiency. However, it is also important to note, contrary reports also exist in the literature (15).

According to the aforementioned role of Vitamin D in reproduction, researchers have tried to assess the association between serum Vitamin D concentrations and assisted reproductive outcomes. In this regard, Pacis et al. (16) in their systematic review titled “Vitamin D and assisted reproduction: should Vitamin D be routinely screened and supplemented prior to assisted reproductive techniques (ART) state that Rudick et al. (17) and Garbedian et al. (18) have confirmed the effect of Vitamin D on improvement of assisted reproductive treatment outcomes, but Aleyasin et al. (19) in their study showed that Vitamin D had no significant effect on outcomes of assisted reproductive treatments. Pacis et al. (16) also stated that in contrary to several reports stating beneficial effect of Vitamin D supplementation on ART outcomes “cost- benefit analysis for a single ART cycle involving fresh single blastocyst embryo transfer suggests that screening and supplementing vitamin D prior to ART might significantly decrease societal cost per ongoing pregnancy by implementing a simple intervention, if the magnitude of the observed effect was confirmed in future studies”. Surprisingly the study by Anifandis et al. (20) conducted on women who were candidates for assisted reproductive treatments showed that the increase in serum level of Vitamin D was associated with decrease in the quality of embryos and the rate of achieving biochemical and clinical pregnancy. Therefore, taking into consideration the controversial results about the effect of Vitamin D supplementation and according to Vanni study (21) stating that the effect of Vitamin D on ART outcomes is not clear and should be evaluated in different populations by randomized controlled trial and cohort studies, hence current trial is very important and valuable.

## Materials and Methods

The clinical trial study was approved by Ethical Committee of Isfahan University of Medical Sciences and was registered in Iranian registry for the clinical trial (IRCT2015111124999N1) and was designed to be carried out at infertile couples that referred to Isfahan Fertility and Infertility Center from March 2016 to June 2016 and candidate of ICSI. Female with age ranging from 18 to 38 years who had Vitamin D level below 30 ng/ml without symptom of Vitamin D deficiency participated in the study.

Based on the ethical committee, initially individuals were questioned regarding clinical symptom of Vitamin D deficiency and individuals with these symptoms were excluded from the study, as Vitamin D treatment was mandatory for these individuals. Additionally, to roll out effects of male factor infertility and advance maternal age, couples with abnormal semen parameter based on WHO (2010) and/or female age greater than 38 were also excluded from the study. Other exclusion criteria were: secondary female infertility, polycystic ovarian disease, endometriosis, congenital or acquired uterine malformations, drugs consumption that would affect metabolism and Vitamin D absorption such as Carbamazepine and Phenobarbital Phenytoin, body mass index of lower 18 or higher 30 kg/m^2^ and hypothyroidism. Couples at risk of ovarian hyper stimulation syndrome or poor endometrium (less than 7 mm or grater 14 mm) were also excluded during the course of the study, since all the embryos for these case were vitrified

Initially 159 couples were interviewed based on Vitamin D level below 30 ng/ml. Fifty one couples were excluded based on exclusion criteria. All of the participants entered the study after giving written informed consent and were allowed to leave the study at any desired time.

**Fig.1:**
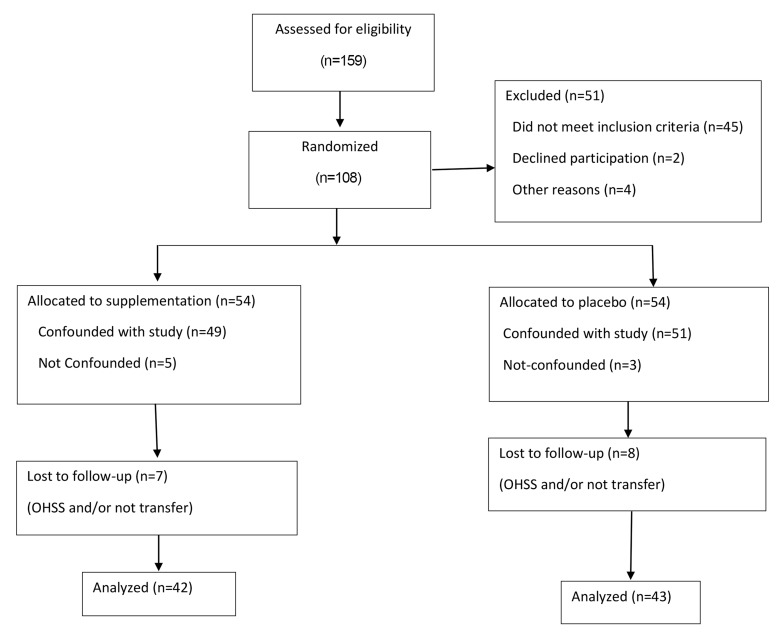
Flow diagram of the progress through the phases of a 2-group parallel randomized trial.

Six couples were also excluded for other reasons including declining to participate. The remaining 108 couples were randomly divided into Vitamin D and placebo groups based computer-generated or random allocation software with one block (Fig .1). Participants or Vitamin D group received a weekly dose of 50000 units of Vitamin D supplementation or placebo for six weeks as pearls orally. Boxes containing Vitamin D and Placebo peals were labeled based on random allocation number, except the two individuals allocating the Vitamin D and placebo, participants, clinician and in vitro fertilization (IVF) laboratory personnel were all blind to the study. Administration of Vitamin D or placebo started on the second day of the last menstrual period (LMP) prior to ICSI cycle and continued to day of hCG administration which was around 6 weeks. Vitamin D (50000 units) and placebo pearls were purchased from Zahravi (Tabriz, Iran).

Serum Vitamin D was assessed by high-performance liquid chromatography and defined based on couple’s information before starting the trial and also six to eight weeks after treatment, on the day of ovum pick. All the Vitamin D assessment was carried out at a single laboratory. The codes were unraveled after completion of data. Semen parameters, including volume, sperm density, percentage motility and normal morphology were also defined based on WHO (2010) manual.

Ovulation induction: all the participants received a combination of recombinant follicle-stimulating hormone (FSH) and human menopausal gonadotrophins (hMG) and were followed by sequential vaginal ultrasound. Gonadotropin releasing hormone (GnRH) antagonist was administered when size of dominant follicles was around 12-14 mm and continued until the day of hCG administration. On the day of hCG administration, number of follicles greater than 12 mm and type of endometrium were also defined and recorded. Type of endometrium was defined according to study by Zhao et al. (22), briefly: cycles were divided into 3 groups depending on the thickness (group 1: =7 mm; group 2: >7 mm to =14 mm; group 3: >14 mm). Each group was subdivided into three groups according to the endometrial pattern as follows: pattern A (a triple-line pattern consisting to a central hyperechoic line surround by two hypoechoic layers); pattern B (an intermediate isoechogenic pattern with the same reflectivity as the surrounding myometrium and a poorly defined central echogenic line); and pattern C (homogenous, hyperechogenic endometrium). Based on exclusion criteria individuals with endometrium thickness of less than 7 mm and grater 14 mm were excluded from the study. Induction of ovulation was induced with administration of 10000 IU hCG when dominant follicles reached size of 17-18 mm. vaginal ultrasound ovum pick up was performed 36 hours post hCG administration. Standard ICSI program was carried out using G-V series (VitroLife, Guttenberg).

Numbers of oocytes were recorded on the day of oocyte retrieval. All the couples underwent ICSI based Isfahan Fertility and Infertility policy. Fertilization rate was calculated based on the number of 2PN observed over the number of injected oocytes. On day 3, embryos were scored for the number of blastomeres, blastomere regularity and percentage cytoplasmic fragmentation. Embryos were considered as "good quality" that had between 6-8 blastomeres with even size and less than 25% fragmentation. These outcomes were taken as primary outcomes.

ß-hCG greater than 20 IU was considered as chemical pregnancy and clinical pregnancy was defined as pregnancy diagnosed by ultrasound through visualization of one or more gestational sac. Of note, multiple gestational sacs were considered as one clinical pregnancy. Therefore, clinical pregnancy rate was defined as the number of clinical pregnancy per 100 embryo transfer. These outcomes were considered as secondary outcomes.

### Statistical analysis

Gathered data were analyzed using SPSS for Windows (version 16, SPSS Inc., Chicago, IL, USA). Continuous variables between two groups were compared with the independent t test, and categorical variables were compared with the chi-square test.

## Results

In the present study, the mean age of women in the intervention group was 31.9 ± 4.2 years and in the control group was 30.8 ± 4.4 years. The mean of body mass index (BMI) in the intervention group was 23.9 ± 2.1 and in the control group was 23.8 ± 1.9 and statistical analysis showed no significant difference between the demographic characteristics and the BMI of the intervention and the control group (P>0.05, Table 1). No statistical difference was observed for male age, educational and duration of infertility and number of previous ART cycles. Therefore, these data suggest that the samples were randomly allocated into the two groups and both groups were similar. We also assess semen parameters between the two groups and no statistical difference was observed between the two groups. Comparison of semen parameters including semen volume, sperm concentration, motility and morphology revealed no statistical differences between the two groups (data not shown).

**Table 1:** Comparison of basal and clinical characteristics of couples in Vitamin D and Placebo groups


Variable		Vitamin D	Placebo	P value

Female age (Y)^*^		31.9 ± 4.2	30.8 ± 4.4	0.29
Male age (Y)^*^		35.26 ± 5.2	35.1 ± 4.7	0.98
Duration of infertility (month)^*^		77.4 ± 22.1	68.1 ± 19.3	0.28
Number of previous ARTcycle^*^		1.9 ± 1.2	2.3 ± 1.5	0.37
Female education (%)				
	High school	12	10	0.45
	Diploma	19	19	
	Master	11	15	
Body mass index (kg/m^2^)^*^		23.9 ± 2.1	23.8 ± 1.9	0.65


ART; Assisted reproductive techniques and *; Data are presented as mean ± SD.

**Fig.2:**
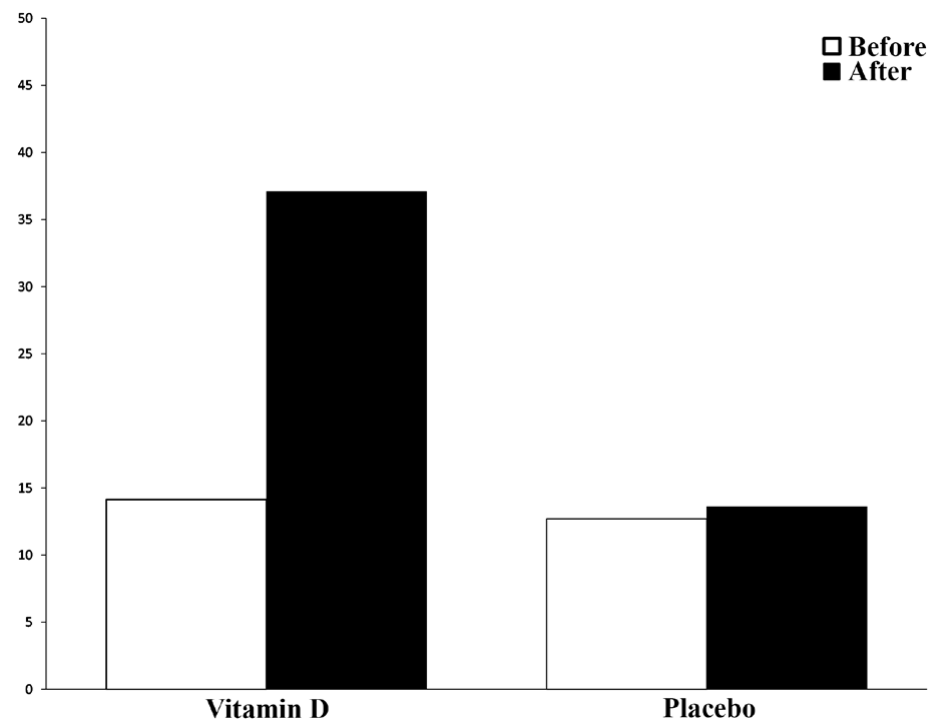
Comparison of serum Vitamin D levels in Vitamin D and placebo groups before and after intervention.

The primary serum Vitamin D levels of the intervention and the control group were 14.4 ± 6.6 ng/ml and 12.7 ± 6.4 ng/ml, respectively. The differences between the two groups were insignificant. Six weeks after treatment with Vitamin D or placebo, the level of Vitamin D significantly raised to 37.1 ± 7.7 ng/ml in the Vitamin D group while it remained low (13.6 ± 6.6 ng/ml) in the placebo group (Fig .2). Unlike in the Vitamin D group, in the placebo group the difference before and after 6 was insignificant.

Regarding the ICSI primary outcomes, Table 2 showed that the mean number of retrieved oocytes in the intervention or Vitamin D group was 9.42 ± 4.4 and in the control group was 8.72 ± 5, and their difference was not statistically significant (P>0.05). Percentage of type A endometrium on the day of hCG injection was 81% and 55.8% in Vitamin D and placebo groups, respectively and the difference between the two groups was statistically significant (P<0.05). The rate of fertilization in the Vitamin D group was 68.80% and in the control group was 68% and the difference was not statistically significant. The rate of good quality embryo on day3 in the Vitamin D group was 59.9 and in the control group was 53.59% and the difference was not statistically significant (P=0.36, Table 2). We also categorize the individuals based on vitamin D deficiency (<10 ng/ml) and insufficiency (10-30 ng/ml) and compared the primary outcomes in the two categories and except for type endometrium, no difference was observed between the two groups for primary outcomes.

**Table 2:** Comparison of ICSI outcomes in Vitamin D and placebo groups


Variable		Vitamin D	Placebo	P value

Endometrium (%)				
	Type A	81	55.8	0.01
	Type B	19	39.5	
	Type C	0.0	04.7	
Mean number of oocyte(Mean ± SD)		9.42 ± 4.4	8.72 ± 5	0.55
Fertilization rate (%)		68.8	68	0.88
Mean number of embryo(Mean ± SD)		5 ± 2.5	4.6 ± 3.3	0.53
Good quality embryo (%)		59.9	53.59	0.36


ICSI; Intracytoplasmic sperm injection.

**Fig.3:**
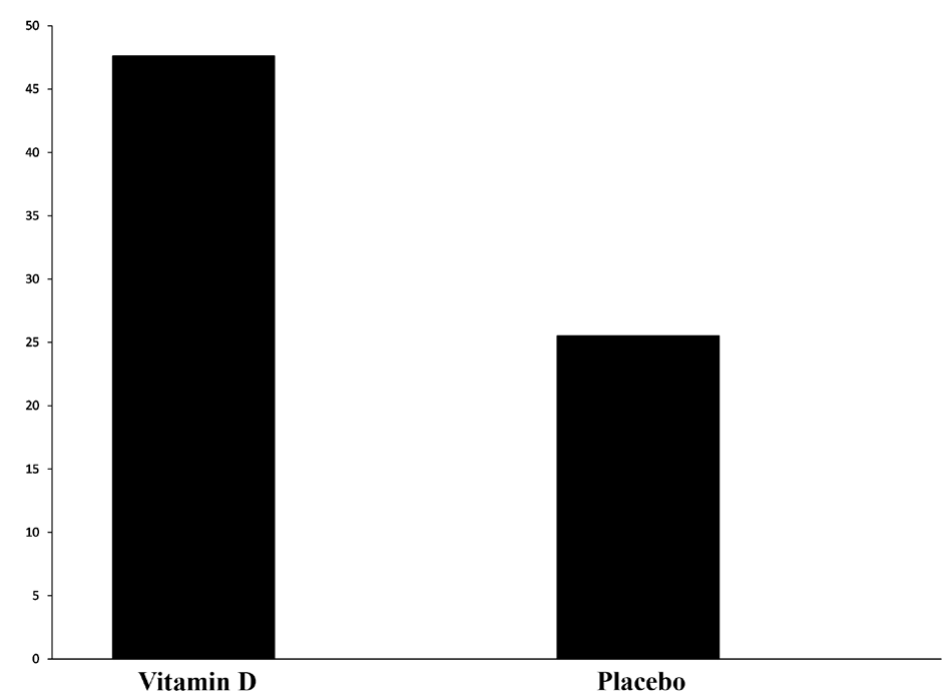
Comparison of chemical pregnancy rate assessed by beta-human chorionic gonadotrophin (ß-hCG) in Vitamin D and placebo group.

**Fig.4:**
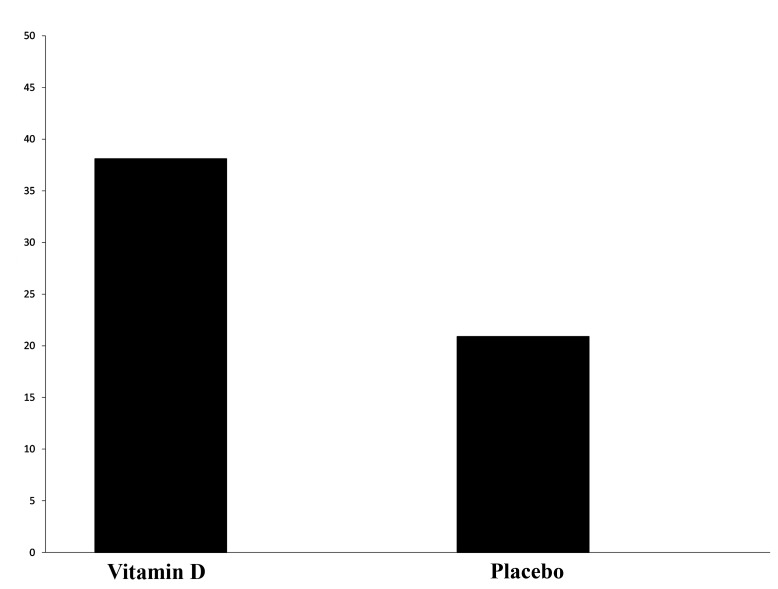
Comparison of clinical pregnancy rate in Vitamin D and placebo groups.

According to the results, chemical pregnancy was defined by positive ß-hCG in the intervention and control groups were 47.6 and 25.5%, respectively, and the difference between both groups was statistically significant (P=0.013, Fig .3). The rate of clinical pregnancy in the intervention group was 38.1% and in the control group was 20.9% and statistical analysis revealed a significant difference between both groups (P=0.019, Fig .4).

## Discussion

Based on background studies Vitamin D plays an imperative role in reproduction and therefore, assessment of Vitamin D and thereby Vitamin D supplementation is becoming part of daily practice. However, role of Vitamin D supplementation during assisted reproductive management remains controversial and there appear to be more room for further study and to evaluate which parameters are most affected by Vitamin D deficiency and thereby supplementation. Part of these controversies may be related to confounding factors affecting both Vitamin D levels and assisted reproductive outcome. An example of these confounding factors is the seasonal effect on Vitamin D level (6), therefore, in this study; the effort was taken so that sampling, measurement of serum Vitamin D level and supplementation took place during spring and early summer. 

The results of this study showed that despite similar demographic and fertility characteristics between the two groups, Vitamin D supplementation significantly improves serum Vitamin D level in comparison to placebo group and this observation is in line with previous studies in this filed (23-25).

Comparing the mean value of serum Vitamin D between both groups before intervention revealed no significant difference between the two groups and 50000 units of Vitamin D supplementation per week for 6 weeks based on the previous study by Diamond et al. (24) resulted in significant increase in serum Vitamin D level compared to before treatment and also compared to placebo group. Indicating that the level of Vitamin D increased to higher than 30 ng/l, the cut of value for Vitamin D deficiency. The outcome of the study is in accordance with previous report Aflatoonian et al. (23) and Spedding et al. (25), indicating that this dosage of Vitamin D supplementation was effective in improving the level of serum Vitamin D.

Our results also reveal that the improved Vitamin D level is also associated with significant difference observed in type of endometrium but no difference was observed between other assessed parameters, including percentage of mature oocytes, fertilization rate and embryo quality. These observations are in concordance with previous report by Asadi et al. (26) and Rudick et al. (17). In this regard, Kinuta et al. (27) show that VDR null mice present uterine hypoplasia. This phenomenon has been related to regulation of expression P450 aromatase activity through *CYP19* gene containing a Vitamin D element in its promoter. These authors state that “the action of Vitamin D on estrogen biosynthesis was partially explained by maintaining calcium homeostasis. However, direct regulation of the expression of the aromatase gene should not be neglected”. But, since, the endometrium in individual undergoing ovarian hyperstimulation is confronted with high level of estrogen in both groups, and the difference in endometrial quality might be due to altered calcium homeostasis in the uterus, but this proposition needs further exploration and validation. It is important to note that as one of the shortcomings of this study, was lack of assessment of estrogen level, but it is also important to consider that we, like others (28) did not observe any difference in the number of follicle and number of oocyte retrieved between the two groups.

Assessment of ICSI outcome in accordance with literature showed that improved Vitamin D has no effect on fertilization and embryo quality on day 3. In contrary to our results and similar studies in this filed, only one study suggest that high concentration Vitamin D reduces embryo quality score following ICSI (20). These authors suggest that glucose provides an essential substrate for cumulus- oocyte complex (COC) and propose that Vitamin D may have a physiological effect on insulin and glucose metabolism in a manner that remains to be elucidated. They believe increase follicular Vitamin D level decreases the availability of glucose to the COC and they state that this proposition may account for negatively correlation with embryo quality and FF Vitamin D levels which opposes our findings and findings of Polyzos et al. (29), Ozkan et al. (28), and Rudick et al. (17) that believe the deleterious effect of Vitamin D deficiency is mediated via on endometrial receptivity rather than reduced embryo quality due to high Vitamin D level. It is important to note the based on their figures number of individuals presenting lower than 15 and 40 ng/ml Vitamin D are very small.

Another major finding of the present study was the difference observed in rates of chemical and clinical pregnancies. In this study rates of chemical and clinical pregnancy rates relative to control group was improved by 10.7% (47.6 vs. 25.5) and 82% (38.1 vs. 20.9), respectively. These results are in accordance with several previous studies, suggests that probably Vitamin D improves ICSI in term of both chemical and clinical pregnancy rates (28-30). Based on the literature and transfer of embryos from donor cycle, it appears that improved effect is very likely related to the improved quality of the endometrium, as also was observed in this study and by other authors (17, 26, 29).

These improved effect has been postulated to be related to mechanisms including i. Miss regulation of NK cell activity, ii. Immunomodulatory role during implantation and recurrent miscarriage, iii. Regulation of cross talk involved between embryos and endometrium which consequently regulates of HOXA10 involved in embryo implantation. It has been shown that endometrial HOX10A expression increase in parallels that Vitamin D receptor around time of implantation, at the time of maximal endometrial differentiation (16, 31). Indeed, increase quality of endometrium, which is reported to be lower in Vitamin D deficient individuals is also related to proper differentiation of endometrial cells (17). 

## Conclusion

Results of the present study showed that consuming Vitamin D supplementation could be effective in improving the clinical outcome of ICSI. Based on literature this effect is very likely to be attributed to local effect of Vitamin D on endometrium.
